# Editorial Notes, News and Miscellany

**Published:** 1913-06

**Authors:** 


### Editorial Notes, News and Miscellany.

---

DR. JOHN TYLER HARRISON, one of the oldest and best known physicians in San Antonio, died in that city May 25 (ult.).

---

DR. A. E. SWEATLAND, late of Little Rock, Ark., has located at Nacogdoches, Texas, and purchased the private hospital of Dr. W. I. M. Smith.

---

DR. FRANK LITTEN, Austin, cranked his car without releasing the clutch. The car ran over him, fracturing the fibula of left leg, compound fracture.

---

TO BE MARRIED June 12th, inst., Annie Laurie Lankford, daughter of Dr. and Mrs. J. S. Lankford of San Antonio, to Frank Pershing Hopwood of that city.

---

DR. H. O. SAPPINGTON of Galveston has been elected Commissioner of Public Health and will have general supervision over all that pertains to sanitation. It is a "big order," but Lr. Sappington is equal to it and will meet all its requirements.

---

A MESSAGE FROM LYDSTON.—As we go to press we receive the following from the modern David who has slain the Octopus—Goliath:

"It may please you to know that we have smashed the machine in Illinois forever and a day." G. FRANK LYDSTON.

---

OUR HANDSOME FRIEND, Dr. M. M. Carrick, is receiving, and justly, much newspaper commendation on his sanitary work throughout the State. He instituted the contest for the cleanest town, and Brownwood received the prize of $500, awarded by Holland's Magazine. Dr. Carrick is now on the editorial staff of the magazine.

